# The *CD177* c.1291A Allele Leads to a Loss of Membrane Expression and Mimics a CD177-Null Phenotype

**DOI:** 10.3390/ijms25052877

**Published:** 2024-03-01

**Authors:** Annalena Traum, Stefanie Jehle, Yannick Waxmann, Anne-Sophie Litmeyer, Heike Berghöfer, Gregor Bein, Reinhard Dammann, Alexander Perniss, Monika Burg-Roderfeld, Ulrich J. Sachs, Behnaz Bayat

**Affiliations:** 1 Institute for Clinical Immunology, Transfusion Medicine and Haemostasis, Medical Faculty, Justus-Liebig-University, 35390 Giessen, Germanyyannick.waxmann@immunologie.med.uni-giessen.de (Y.W.); anne-sophie.litmeyer@immunologie.med.uni-giessen.de (A.-S.L.); heike.berghoefer@immunologie.med.uni-giessen.de (H.B.); gregor.bein@immunologie.med.uni-giessen.de (G.B.); 2 Institute for Genetics, Faculty of Biology and Chemistry, Justus-Liebig-University, 35390 Giessen, Germany; reinhard.dammann@gen.bio.uni-giessen.de; 3 Institute for Anatomy and Cell Biology, German Center for Lung Research, Excellence Cluster Cardio-Pulmonary Institute (CPI), Justus-Liebig-University, 35392 Giessen, Germany; aperniss@bwh.harvard.edu; 4 Faculty of Biology and Chemistry, Fresenius University of Applied Sciences, 65510 Idstein, Germany; 5 Department of Thrombosis and Haemostasis, Giessen University Hospital, 35390 Giessen, Germany

**Keywords:** CD177, c.787A>T, c.1291G>A, PR3

## Abstract

CD177 is a glycosyl phosphatidyl inositol (GPI)-linked, neutrophil-specific glycoprotein that in 3–5% of normal individuals is absent from all neutrophils. The molecular mechanism behind the absence of CD177 has not been unravelled completely. Here, we analyse the impact of the recently described *CD177* c.1291G>A variant on CD177 expression. Recombinant *CD177* c.1291G>A was expressed in HEK293F cells and its expression on the cell surface, inside the cell, and in the culture supernatant was investigated. The *CD177* c.1291G>A protein was characterised serologically and its interaction with proteinase 3 (PR3) was demonstrated by confocal laser scanning microscopy. Our experiments show that *CD177* c.1291G>A does not interfere with CD177 protein biosynthesis but affects the membrane expression of CD177, leading to very low copy numbers of the protein on the cellular surface. The mutation does not interfere with the ability of the protein to bind PR3 or human polyclonal antibodies against wild-type CD177. Carriers of the c.1291G>A allele are supposed to be phenotyped as CD177-negative, but the protein is present in soluble form. The presence of *CD177* c.1291A leads to the production of an unstable CD177 protein and an apparent “CD177-null” phenotype.

## 1. Introduction

CD177 is a glycosyl phosphatidyl inositol (GPI)-linked, neutrophil-specific glycoprotein that is expressed on the plasma membrane of circulating neutrophils, often with a bimodal pattern with positive and negative subpopulations [1]. It has been shown that CD177 binds PR3 on the neutrophil surface [2] and that the CD177-PR3 complex contributes to the enhanced migration of the CD177-positive neutrophil subset [3].

In 3–5% of the normal population, CD177 is absent from all neutrophils, and these individuals are usually denoted as CD177-null [4]. It is uncertain whether CD177-null individuals are susceptible to certain diseases, but CD177-positive neutrophils seem to suppress tumorgenesis in epithelial cells and improve survival in patients with colorectal cancer [5]. The epitope of human neutrophil antigen 2 (HNA-2) is located on CD177. Exposure of CD177-null individuals to CD177 such during pregnancy, can induce the formation of anti-CD177 (i.e., anti-HNA-2) isoantibodies. These antibodies play a role in neonatal alloimmune neutropenia (NAIN) when transported to the baby through the placenta [6]. The binding of anti-HNA-2 to target antigens expressed on neutrophils leads to the elimination of these cells from the bloodstream, resulting in neutropenia in the fetus. Similarly, anti-CD177 antibodies, when present in blood components, can induce transfusion-related acute lung injury (TRALI). The binding of transfused antibodies to CD177 on the recipient’s neutrophils activates the neutrophils and leads to degranulation and ROS production. This process impairs endothelial barrier function and causes pulmonary oedema associated with TRALI [7].

The molecular mechanism behind the absence of CD177 has not yet been unravelled completely. A point mutation *CD177* c.787A>T in exon 7 induces a premature stop-codon and was initially identified as a major cause of the CD177-null phenotype [8]. Subsequent studies however have demonstrated that only 40% of CD177-null mothers with anti-CD177 in their plasma and a newborn with NAIN were homozygous for this mutation [9]. Further analysis led to the identification of a second polymorphism, CD177 c.1291G>A. This mutation alters the GPI-anchor sequence of CD177, and it has been speculated that this mutation may affect membrane stability [10].

In the present study, we aimed to analyse the *CD177* c.1291G>A variant with respect to expression, PR3 interaction, and immunological consequences.

## 2. Results

### 2.1. Characterisation of a Soluble Variant of CD177

**Figure 1:**
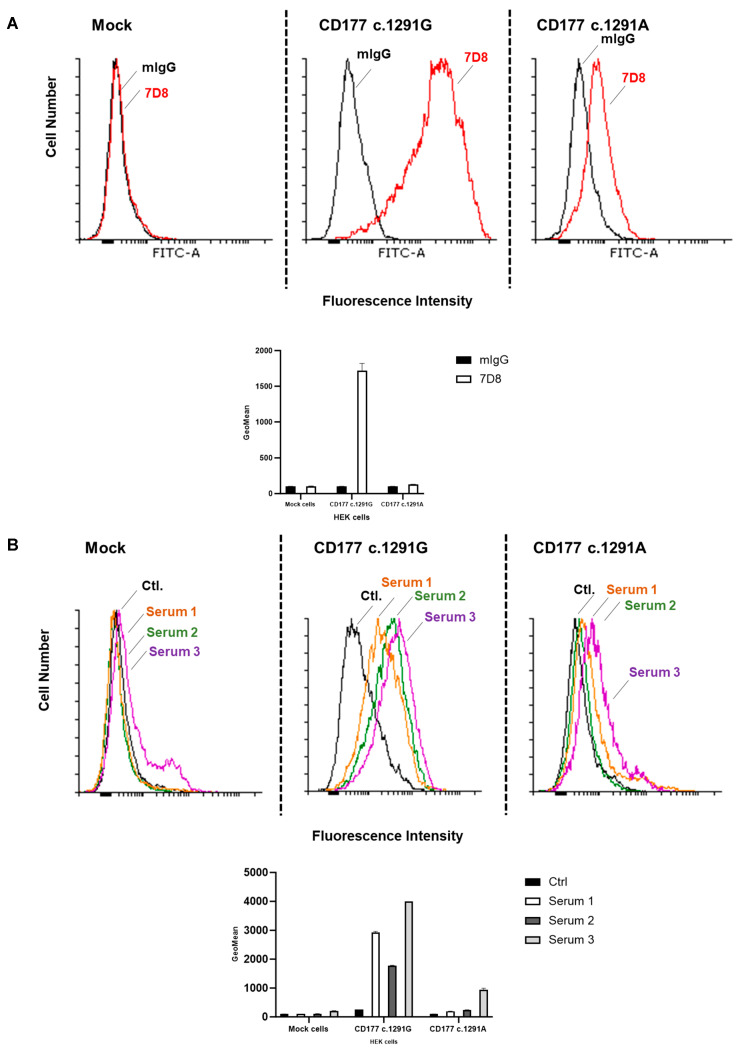
Characterisation of stably transfected HEK293F cells expressing CD177 c.1291G (wildtype) or *CD177* c.1291A (mutant) by flow cytometry. (**A**) HEK293F expressing CD177 c.1291G or CD177 c.1291A were incubated with murine moab 7D8 against CD177 or mIgG (as isotype control). After washing, bound antibodies were detected using Alexa Fluor^®^ 488-conjugated donkey anti-mouse IgG. Mock-transfected cells were used as negative control. (**B**) The binding of human anti-CD177 (obtained from immunised *CD177* c.787T mothers (*n* = 3) to CD177 wildtype and c.1291A mutant was evaluated in FACS. Stably transfected HEK293F cells expressing wildtype CD177 or the CD177 mutant were incubated with sera containing anti-CD177 isoantibodies. Bound human IgGs were detected with FITC-conjugated goat anti-human IgG and analysed by flow cytometry. Results are presented as flow cytometry histogram (upper panel) and bar histogram (lower panel).

The expression of CD177 proteins (*c.1291G* and *A* alleles) on the cell surface and in the cytoplasm of transfected cells was evaluated. Flow cytometry analyses using 7D8 moab showed a positive reaction with transfected HEK cells expressing *CD177 c.1291G*. In contrast, *CD177* c.1291A mutant cells gave only weak signals (Figure 1A). Comparable results were obtained when using MEM166. A comparable reaction pattern was also observed with human polyclonal sera containing anti-HNA-2 antibodies obtained from *CD177 c.787T* mothers: they gave clearly positive reactivity with *CD177 c.1291G*, but only weak or no reactivity with *CD177* c.1291A cells (Figure 1B). These results indicate a reduced expression of *CD177* c.1291A on the cell surface or a significant structural change that impairs the binding of anti-CD177 antibodies.

**Figure 2:**
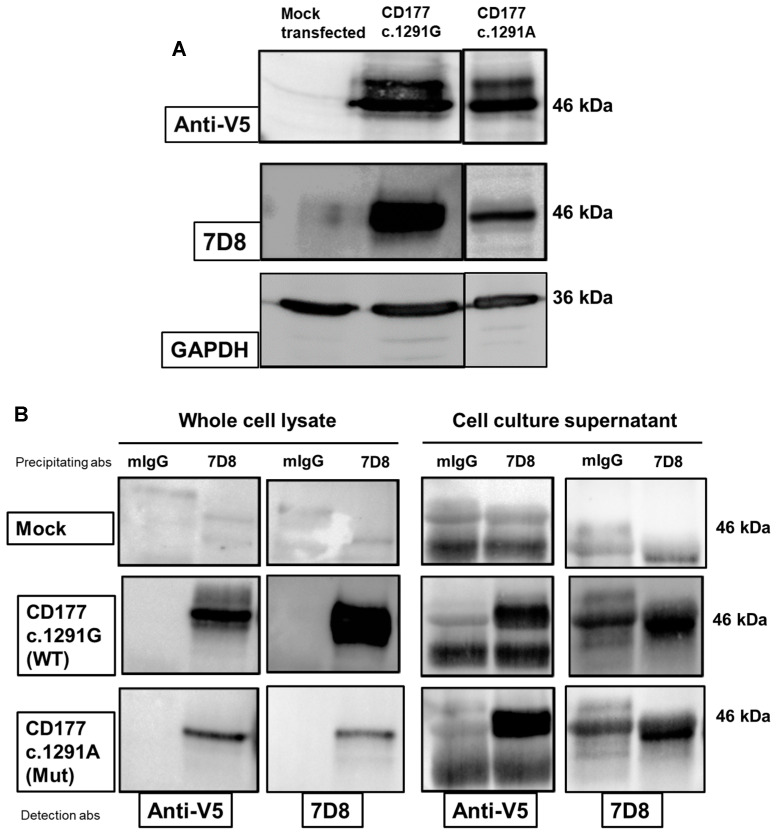
Characterisation of *CD177* c.1291G and *CD177* c.1291A proteins in immunoblot and immunoprecipitation. (**A**) Immunoblot analysis of cell lysate from CD177 transfected (wildtype or c.1291A mutant) and untransfected HEK293F cells. Proteins in cell lysate of transfected and untransfected HEK293F cells were immobilised and incubated with 7D8 or anti-V5 or GAPDH (as internal control). Bound antibodies were visualised using horseradish peroxidase (HRP)-conjugated donkey anti-mouse IgG. (**B**) CD177 proteins from cell lysates (left panel) or cell culture supernatants (right panel) of stably transfected HEK293F cells expressing CD177 proteins (wildtype or c.1291A mutant) were precipitated using 7D8 or mIgG (as control). Separated proteins were then visualised either with 7D8 or with anti-V5 moabs. Equal amounts of total protein were used in these experiments. Figures are representative of three independent experiments.

In order to detect intracellular CD177, immunoblot analyses of cell lysate and culture supernatant using moabs 7D8 and anti-V5 (against the plasmid protein tag) were performed. Anti-V5 detected a band of approximately 46 kDa in cell lysates of both *CD177* c.1291G or c.1291A transfected HEK293F cells, indicating comparable expression of CD177 wildtype and mutant in transfected HEK293F cells. In contrast, 7D8 reacted strongly with *CD177* c.1291G, but detected only a weak band in *CD177* c.1291A, consistent with an alteration of the 7D8 epitope induced by the c.1291A substitution (Figure 2A).

Since 1291G>A polymorphism is located next to the GPI-anchor sequence, we hypothesized an effect of c.1291G>A on membrane anchoring. To further assess this hypothesis, CD177 was precipitated from cell lysates and cell culture supernatants and detected with anti-V5 and 7D8 moabs (Figure 2B). Comparable to the findings in the immunoblot, 7D8 precipitated *CD177* c.1291G much stronger than *CD177* c.1291A from cell lysate. The difference in intensity of detected bands in both cells might be the result of the distinct binding capacity of 7D8 monoclonal antibody moab to *CD177* c.1291G and c.1291A forms. No band was detected in HEK293F cells transfected with mock plasmids. However, since immunoprecipitation with mIgG as a negative control leads to the precipitation of a band that is almost the same size (45–46 kDa) as CD177, the interpretation of CD177 in the supernatant is difficult. This band is also precipitated from mock cells, indicating a cross-reactivity of mIgG with a protein expressed in HEK cells.

Similar results were observed when CD177 proteins were precipitated from culture supernatants (Figure 2B, right panel). While no specific proteins could be precipitated using the supernatant of mock cells, anti-V5 detected 7D8-precipitated proteins in comparable intensity from the culture supernatant of both wildtype as well as mutant cells, indicating a significant release of CD177 proteins in the supernatant. These data support our assumption that the c.1291G>A polymorphism affects the membrane stability of CD177 so that almost all *CD177* c.1291A is found in soluble form and not on the cell surface.

### 2.2. Correlation between Soluble CD177 in Serum and c.1291 Alleles in CD177 Gene

**Figure 3:**
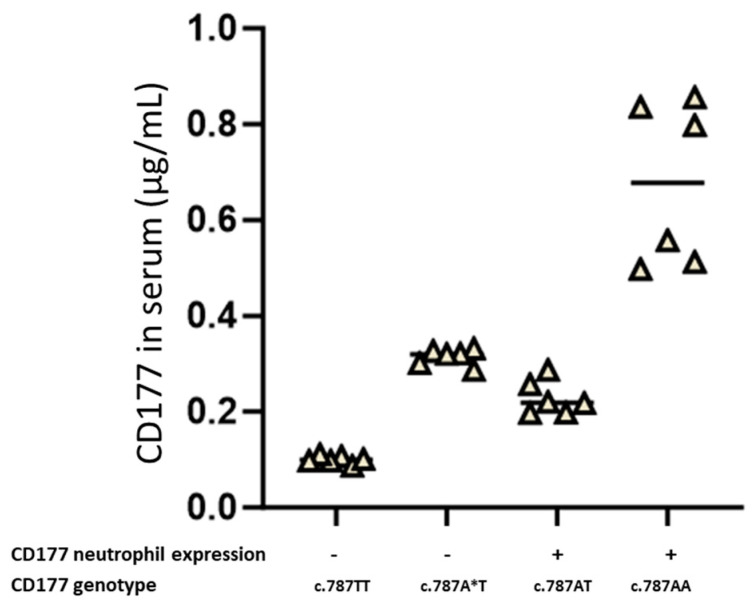
Analysis of membrane-bound and soluble CD177 (sCD177) in the serum of *CD177* c.787A>T genotyped donors. CD177-phenotyped donors (*n* = 6 for each group) were genotyped for the *CD177* c.787A>T polymorphism and then the concentration of soluble CD177 protein in serum was determined by sandwich ELISA as described. A and A* are assigned for CD177 gene c.1291G and c.1291A alleles, respectively. The median of three independent experiments is shown.

According to previous data, individuals in a normal population can be categorised into two groups based on CD177 expression on neutrophils: CD177-negative (no neutrophils express CD177, 0%) and CD177-positive (neutrophils express CD177 on the surface; Figure 3). The *CD177* c.1291G>A polymorphism affects the expression of CD177 on neutrophils and in serum; c.787T/c.787A (AT) individuals without c.1291A allele express CD177 on the neutrophil surface and are therefore phenotyped as CD177-positive. Individuals with a *CD177* c.787T/c.787A genotype with c.1291A (A*T) allele are phenotyped as CD177-negative but express soluble CD177 in plasma. In contrast, no soluble CD177 was detected in the serum of *CD177* c.787T/c.787T individuals with no CD177 surface expression. The largest amount of CD177 was detected in the serum of individuals with a *CD177* c.787AA genotype, which does express CD177 on their neutrophils. This could explain the *CD177* gene dosage effect (Figure 3). With these results, individuals who do not express CD177 on their neutrophil surface can be divided into two groups: those with soluble CD177 present in their serum, and those who do not have soluble CD177 in their plasma.

### 2.3. Reactivity of sCD177 Variants with Human Anti-CD177 Antibodies

**Figure 4:**
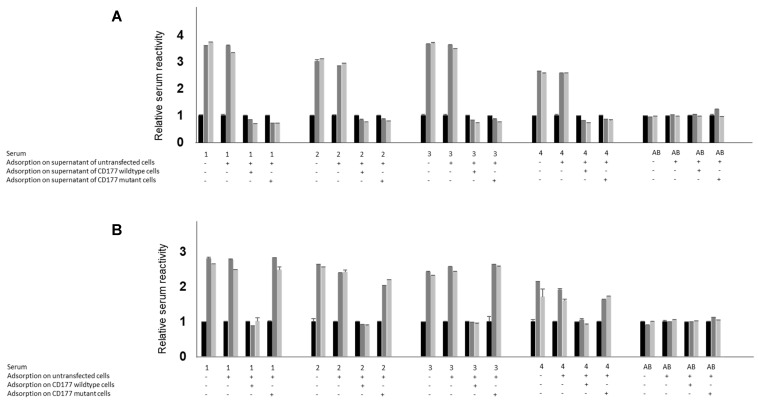
Analysis of anti-HNA-2 isoantibodies reactivity with immobilised CD177 (wildtype or c.1291A mutant) protein in ELISA. Proteins from cell culture supernatants of transfected cells, either mock (black), wildtype (dark grey), or mutant (light grey), were immobilised on V5-pre-coated microtiter plate. Before adding to immobilised protein, sera were first incubated with supernatant obtained from untransfected HEK293F cells (−/−), followed by adsorption to supernatant from HEK293F cells expressing CD177 wildtype (−/+G) or supernatant from *CD177* c.1291A mutant (−/+A). After washing, human serum containing anti-CD177 antibodies (from *CD177* c.787T individuals) or control serum (as negative control) was added. Bound human IgGs were detected using horseradish-labelled goat anti-human IgG. (**A**) To differentiate the reactivity of human antibodies against CD177 wildtype and mutant expressed on cell surface, sera were first incubated with untransfected HEK293F cells (−/−), followed by adsorption to HEK293F cells expressing either CD177 wildtype (−/+G) or CD177 *c.1291A* mutant (−/+A). In similar experiments, adsorption of human antibodies to proteins present in supernatant was conducted (**B**). The adsorbates were added to immobilised CD177 protein (either mock (black), wildtype (dark grey), or mutant (light grey)) and bound human IgG was detected using horseradish peroxidase-labelled goat anti-human IgG, (*n* = 4). Values are presented as mean ± SD (*n* = 5) (cut-off determined for supernatant ELISA was 1486 as determined with sera from non-immunised healthy donors with blood group AB (*n* = 20). The statistical significance of the difference in adsorption between mutant and wild-type CD177 was analysed by ANOVA using GraphPad Prism 7.

Soluble CD177 proteins from the supernatant of transfected HEK293F cells were immobilised on anti-V5 coated wells. Using HRP-labelled 7D8, comparable reactivity could be demonstrated for both variants (Figure 4). Notably, all 4 human sera containing anti-HNA-2 obtained from *CD177* c.787T mothers gave comparable reactivity with both variants. To further differentiate the reactivity of anti-HNA-2, an adsorption strategy was conducted. Anti-HNA-2 sera were adsorbed to transfected cells expressing *CD177* c.1291G and then tested against both variants of immobilised sCD177. All sera became non-reactive with both variants following the adsorption step (Figure 4A; *n* = 4, *p* < 0.001 for *CD177* c.1291G and c.1291A, respectively). In contrast, when anti-HNA-2 sera were adsorbed to cells transfected with *CD177* c.1291A, their reactivity did not change and they remained positive (unspecific loss of reactivity by an average of 10% after adsorption using cells; *p* = 0.1855 for adsorption using *CD177* c.1291A; and *p* < 0.001 for adsorption using *CD177* c.1291G). This finding indicates that there is only a low copy number of the c.1291A allele on the membrane of transfected cells or it is completely absent. In another set of experiments (Figure 4B), sera were adsorbed to both variants of soluble CD177. Either of the two soluble proteins led to non-reactivity of the adsorbate with both, *CD177* c.1291A and c.1291G. Taken together, these results indicate the presence of *CD177* c.1291A in the cell culture supernatant, but its elimination from the cell surface. The serum adsorption with cell culture supernatants demonstrates that both variants are detectable in soluble form, and that the soluble forms of both have comparable epitope characteristics, since both variants can fully adsorb human polyclonal antibodies.

### 2.4. Co-Localisation Analysis by Confocal Laser Scanning Microscopy

**Figure 5:**
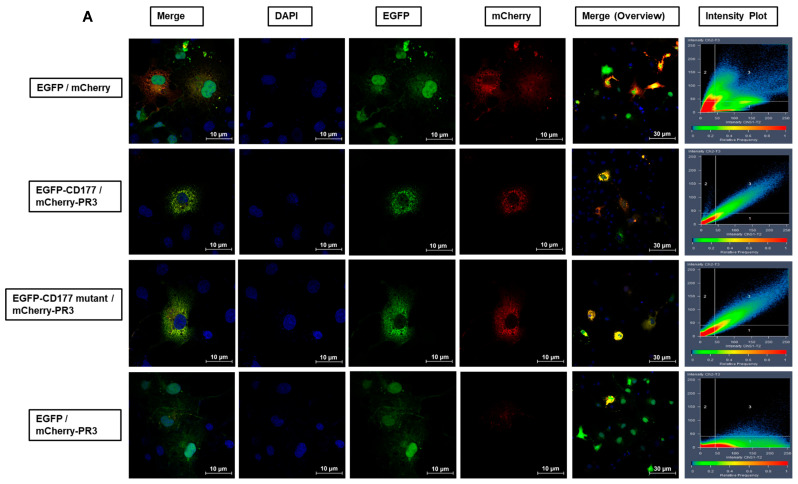

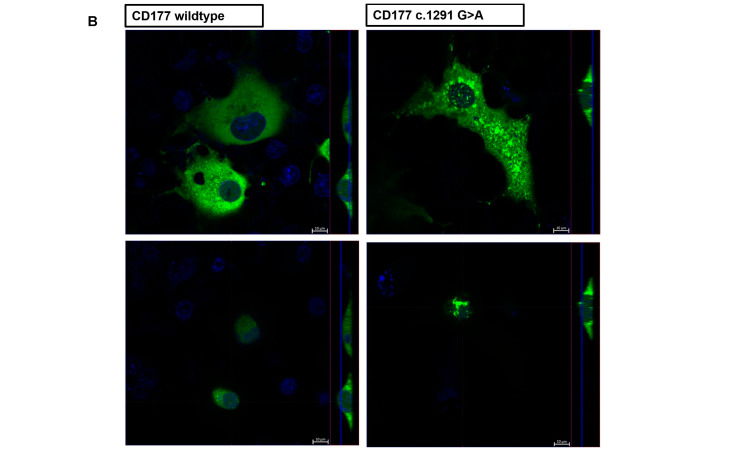
Visualisation of PR3 co-localisation with CD177 wildtype or mutant by confocal laser scanning microscopy. (**A**) COS-7 cells were cultured on cover glasses and were double transfected with the green fluorescent vector pEGFP-CD177 wildtype or mutant (*CD177* c.1291A) and the red fluorescent vector pcDNA3.1/mCherry-PR3. As controls, COS-7 cells were transfected with the empty vectors pEGFP and pcDNA3.1/mCherry or pEGFP together with pcDNA3.1/mCherry-PR3. Cell nuclei were stained with DAPI. Fluorescent signals were examined by the use of laser scanning confocal microscopy. EGFP signals appear in green, mCherry signals appear in red, and nuclear staining with DAPI appears in blue. Where both proteins are co-localised, fusion signal appears to be yellow. The last column presents the intensity plot of red and green co-localisation generated by ZEN software. Quadrant 1 shows the EGFP signal, quadrant 2 shows the mCherry signal and quadrant 3 shows the population with both signals visible. Overview images were taken at 25-fold magnification, detailed images at 63-fold magnification. Presented images are representative for *n* = 20 different analyses per transfection (**B**) To visualise the surface expression of CD177 proteins (wildtype and mutant), live cell imaging was performed. Live single transfected cells expressing EGFP-CD177 proteins (wildtype and mutant) were stained using Hoechst 33342. To visualise the cell surface, orthogonal sections were created through the cells from the z-stack image. The lines drawn in red show the *x*-axis, green the *y*-axis, and blue the *z*-axis. The upper panels (left and right) show the basal plane (*z*-axis 5) and the lower panels show the apical plane (*z*-axis 11) of the CD177 wildtype cells and *CD177* c.1291G>A, respectively. At the bottom right, the apical plane shows a homogeneous distribution, while D shows a heterogeneous distribution with conglomerates of EGFP-CD177, which is shown in the Z-X plane. EGFP-CD177 wildtype transfected cells showed surface expression of the fusion proteins while EGFP-CD177 mutant c.1291G>A showed surface expression in the form of conglomerates which was unstable on the cell membrane (magnification 63×). All figures are representative of three independent experiments. To visualise the surface expression of CD177 proteins (wildtype and mutant), live cell imaging was performed. Living single transfected cells expressing EGFP-CD177 proteins (wildtype and mutant) were stained using Hoechst 33342. To make the cell surface visible, orthogonal cuts through the cells from z-stack imaging were generated. The lines drawn in red show the *x*-axis, green the *y*-axis, and blue the *z*-axis. A and C show the basal plane (*z*-axis 5) and B and D show the apical plane (*z*-axis 11) of the CD177 wildtype cells respective *CD177* c.1291G>A. B The apical plane shows a homogenous distribution, whereas D shows a heterogenous distribution with conglomerates of EGFP-CD177.

CD177 and PR3 are co-localised on the surface of neutrophils [2]. The co-localisation has been reported to regulate the proteolytic activities of PR3 [3]. To investigate whether the *CD177* c.1291A variant is able to bind PR3, CD177 co-transfection with PR3 was conducted and cells were analysed by confocal laser scanning microscopy (CLSM; Figure 5). Visualisation of EGFP-CD177 signals together with mCherry-PR3 signals show the expression of both proteins in physical closeness in transfected cells (Figure 5A, second row). Importantly, EGFP-*CD177* c.1291A together with mCherry-PR3 gave comparable signals (Figure 5A, third row). As expected, a correlation between the red and green signals was absent in the control experiments (first and last row). Further analysis of membrane stability in CD177 wildtype and variant using live imaging (63× magnification) demonstrated surface expression of the EGFP-CD177 wildtype fusion protein (Figure 5B). In contrast, the EGFP-CD177 variant showed a surface expression in the form of conglomerates, which quickly disappeared from the surface (Figure 5B; representative images are shown).

Statistical analysis was performed for the data revealed by ZEN software (Table 1). Correlation R as a mathematical description of the dependence was significantly higher in CD177 (wildtype or variant)/PR3 co-transfected cells in comparison to controls (0.69 and 0.66 versus 0.10 and 0.32; *p* < 0.05 for all comparisons), and CD177 wildtype and CD177 variant showed similar R values (0.69 vs. 0.66, *p* = 0.65).

## 3. Discussion

The molecular mechanism underlying the absence of CD177 is still unclear, which has implications for our understanding of CD177 physiology, but also for diagnostic testing in affected individuals. Two mutations have been described so far. Only about 40% are homozygous for the *CD177* c.787A>T point mutation [9], which leads to a premature stop codon. A second mutation associated with the absence of CD177 from the neutrophil surface is *CD177* c.1291G>A [10], but its molecular impact on CD177 has not been studied so far. Here, we show that *CD177* c.1291G>A does not interfere with CD177 protein synthesis and does not affect its interaction with PR3, but does affect CD177 expression on the cell surface.

This observation has numerous implications. First, our data can explain why CD177 is not found on the neutrophil surface in *CD177* c.787T/c.1291A heterozygous individuals, as one allele (c.787T) is non-functional and the other allele (c.1291A) is not expressed on the cell membrane. The lack of stable membrane expression could be due to this variant being released from the membrane by the absence of the GPI anchor. The SNP c.1291G>A (p.Gly431Arg) is located within the carboxyl-terminal hydrophobic region of CD177 GPI-signal modules. The change of the residue from 431Gly (1291G allele) to 431Arg (1291A allele) significantly alters the hydrophobicity since arginine has a hydrophilic side chain while glycine has an uncharged side chain. Therefore, 431Arg produces a pro-peptide with an unfavourable GPI-attachment signal, which destabilises CD177 on the cell membrane [11]. Accordingly, *CD177* c.787T/c.787A heterozygous individuals who carry the *CD177* c.1291A allele have soluble CD177 in their serum, whereas *CD177* c.787T/c.787A heterozygous individuals with a *CD177* c.1291G allele express CD177 on the surface of their neutrophils.

This is also evident from a comparable amount of wildtype and variant CD177 inside transfected cells, but a sharp difference in the amount of CD177 found on cell surfaces. Analysis of the two protein sequences using Big PI prediction software (version stand: 05.17.2005) also showed that the *CD177* c.1291A variant was unlikely to have a GPI structure (Supplementary Figure S1). Second, our observation leads to a peculiarity previously unknown in immunohaematology: the absence of an immunogenic protein from the neutrophil surface (“CD177-null”) with the simultaneous presence of the same protein in the plasma. As we demonstrated in adsorption studies, both CD177 proteins (wild-type and variant) were able to fully adsorb anti-HNA-2 antibodies from human sera, indicating that both proteins carry all relevant epitopes against which anti-HNA-2 are usually directed. Accordingly, since CD177 is only absent from the neutrophils, but not from the plasma in individuals carrying *CD177* c.1291A, we believe these individuals are unable to become immunised against CD177. In fact, all immunised cases documented in our laboratory (*n* = 9) were *CD177* c.787T homozygous and none had the *CD177* c.787A/c.1291A genotype. Fourteen of the total 15 individuals with HNA-2 isoantibodies analysed in a recent international study, including 9 immunised mothers, 3 immunised donors, and 3 other patients with HNA-2 isoantibodies, were homozygous for the *CD177* c.787T allele. Only one NIN mother was heterozygous for the *CD177* c.787A>T mutation but also negative for c.1291G>A mutation, and it was unclear why CD177 expression was absent from her cells [9].

Third, membrane expression of PR3 is mainly mediated by CD177 [12]. Our analysis of co-transfected cells expressing EGFP-labelled CD177 wild-type or c.1291A variant proteins along with mCherry-labelled PR3, indicated an identical co-localisation of PR3 with both, CD177 wild-type and variant. This suggests that the protein encoded by *CD177* c.1291A still binds to PR3. It has been demonstrated that both proteins interact in their soluble form in vitro and it was speculated that CD177 may function as a protective agent that down-regulates the enzymatic activity of PR3 [7,13]. Although the conclusion of co-localisation of PR3 with *CD177* c.1291A was based on a comparison of co-localisation of wildtype CD177 with PR3 (as a known fact), given the limitations of the fluorescence microscopy technique for inferring comparable co-localisation of *CD177* c.1291 A with wild-type CD177, alternative techniques such as surface plasmon resonance analysis of PR3 as a ligand for immobilised *CD177* c.1291A or wildtype are required to support this conclusion. Whether or not soluble CD177 can efficiently bind PR3 to support PR3-associated functions of neutrophils, is unknown. We have previously shown that transfused CD177/PR3 complexes present in blood components may become adsorbed to PECAM-1 on activated endothelial cells. Here, they can trigger endothelial barrier dysfunction and transfusion-related acute lung injury TRALI in anti-HNA-2 antibody carriers [13]. We are currently unable to state whether variant CD177/PR3 is also able to interact with PECAM-1, but data obtained in the study presented here have not brought evidence that would argue against it.

The second and third observations are of potential medical relevance since individuals phenotyped as CD177-null may in fact carry *CD177* c.787T and c.787A/1291A. Since these individuals produce soluble CD177, they will not become immunised against CD177; and their plasma may contain relevant amounts of CD177.

The data presented in this study comes with some limitations. In particular, whether or not *CD177* c.1291A affects CD177/PR3-associated functions cannot be answered and needs to be analyzed further. In addition, the frequency of *CD177* c.1291A in the normal population and in various ethnicities should be investigated.

## 4. Materials and Methods

### 4.1. Serum Samples

Serum samples used for this study were obtained from mothers who had a child with NAIN and from female blood donors with a history of pregnancy who were screened for the presence of anti-granulocyte antibodies. All individuals were white Europeans. Anti-CD177 (anti-HNA-2) isoantibodies from individuals homozygous for the *CD177* c.787T allele (*n* = 6) were identified and characterised in the granulocyte immunofluorescence test (GIFT) [14]. Sera from healthy blood donors with blood group AB were collected as controls. All subjects gave their informed consent for inclusion before they participated in the study. The study was conducted in accordance with the Declaration of Helsinki.

### 4.2. CD177 Phenotyping

Neutrophils were isolated from EDTA-anticoagulated blood from healthy donors (*n* = 10) by dextran sedimentation (Dextran 500, Roth, Karlsruhe, Germany). The expression of CD177 on the surface of neutrophils was determined by flow cytometry using monoclonal antibodies (moabs) against CD177 (clone MEM166) or mouse (m) IgG (as isotype control). The percentage of CD177 expression on the neutrophils was determined for each donor. Accordingly, donors were categorised into two groups: >0% (with CD177 expression on the neutrophil surface) or 0% (no CD177 expression on the neutrophil surface).

### 4.3. CD177 Genotyping

DNA was isolated from CD177-phenotyped subjects and the *CD177* gene was amplified using a long-range PCR strategy. For the c.787A>T and c.1291G>A polymorphisms, *CD177* exon 7 and exon 9, respectively, were sequenced with a sequencing kit (BigDye v3.1, Applied Biosystems, Waltham, MA, USA) on a DNA analyser (ABI 3730xl, Applied Biosystems) using primers (5′-TCTTTGGGCCCCACACTAAACA-3′) for the SNP c.787A>T and primer 5′-AGGTTG AGTGTGGGGGTGGTCAGC-3′ for SNP c.1291G>A) as previously described [10].

### 4.4. Detection of Soluble CD177 in Serum

Soluble CD177 in serum (from mothers with NAIN children and healthy donors) was detected using a sandwich ELISA as previously described [13]. The microtitre wells were coated overnight at 4 °C with moab against CD177 (clone MEM166, 5 µg) in buffer. After washing, 100 µL of serum from CD177-phenotyped donors and NAIN mothers were added and incubated at 4 °C for 1 h. After washing the wells with phosphate-buffered saline (PBS; Oxoid, Hampshire, UK) supplemented with 0.02% bovine serum albumin (BSA; Ortho Clinical Diagnostics, Neckargemünd, Germany) 9 µg biotin-labelled moab against CD177 clone 7D8 (the antibody-producing hybridoma cells were a generous gift from Dr. D. Stroncek, NIH, Bethesda, ML, USA, and 7D8 moab was purified from the cell culture supernatant of hybridoma cells using an in-house technique) was added to detect immobilised CD177 and incubated for 1 h at room temperature. To detect bound anti-CD177, 100 µL of horseradish peroxidase-labelled anti-human IgG (dilution 1:50,000, GE Healthcare, Chicago, IL, USA) was added. After washing, the wells were incubated for one hour at room temperature. Bound antibodies were detected with 3,3′,5,5′-tetramethylbenzidine substrate solution (Sigma Rowville, Clayton, Australia). The reaction was stopped with 100 µL of 1.0 mol/L HCl and read at 450 nm on an ELISA reader (Tecan).

### 4.5. Cell Lines

HEK293F cell lines were a generous gift from Professor Bernhard Nieswandt, Institute for Experimental Biomedicine, Wuerzburg, Germany. Other cell lines were purchased from the American Type Culture Collection (ATCC).

### 4.6. Expression of CD177 on HEK293F Cells

Expression plasmids containing full-length *CD177* carrying the c.1291A allele were generated from wildtype *CD177*. In brief, pcDNA3.1/V5-His^©^ vector (Thermo Scientific, Waltham, MA, USA) containing cDNA sequence of wildtype *CD177* was mutated by the use of mutagenic primers 5′-CCA GCG CTG TGG TGG AGA GTG GTT TGC CCT TG-3′ and 5′-CAA GGG CAA ACC ACT CTC CAC CAC AGC GCT GG-3′ using QuikChange II Site-directed mutagenesis kit (Agilent, Santa Clara, CA, USA). HEK293F cells were grown in DMEM medium (Capricorn Scientific, Ebsdorfergrund, Germany) supplemented with 10% fetal calf serum (FCS; PAN-Biotech, Germany), Penicillin/Streptomycin (P/S; PAN-Biotech, Aidenbach, Germany) and were transfected with the vectors containing the cDNA of the wildtype or c.1291A mutant human CD177 using Effectene (QIAGEN, Hilden, Germany) or jetOPTIMUS (Polyplus-transfection, Illkirch, France), as recommended by the manufacturer. Transfected cells were selected with geneticin at a final concentration of 800 µg/mL (GIBCO BRL, Waltham, MA, USA) and later maintained with 400 µg/mL geneticin.

#### Characterisation of Stably Transfected HEK293F

Aliquots of 2.5 × 10^5^ CD177 transfected (*CD177* wildtype or c.1291A) or mock-transfected HEK293F cells (as a control) were washed with PBS/BSA 0.2% and centrifuged at 6900× *g*. for 1 min. After washing, the cells were incubated with 0.4 µg of 7D8 moab for 30 min at 4 °C. Purified mIgG (Ancell Corporation, Bayport, MN, USA) was used as an isotype control. The bound antibodies were detected using Alexa Fluor™488-conjugated donkey anti-mouse IgG (dilution 1:50; Life Technologies, Waltham, MA, USA). To further characterise the surface expression of CD177 on HEK293F cells, in some tests, transfected cells were incubated with 30 µL serum containing anti-CD177 isoantibodies for 1 h at 37 °C. After washing with 0.2% PBS/BSA, cells were stained with 50 µL FITC-conjugated goat anti-human IgG (dilution 1:50; Jackson Immuno Research, West Grove, PA, USA) for 30 min at 4 °C. Labelled cells were washed again, resuspended in Cellfix buffer (Becton Dickinson, Franklin Lakes, NJ, USA) and analysed by flow cytometry (BD FACS Canto II; Franklin Lakes, NJ, USA). A total of 10^5^ cells were analysed per experiment. The presence of CD177 proteins (wildtype and mutant) in culture supernatant and cell lysate was evaluated by immunoblot. Whole-cell lysates were prepared. A total of 10^7^ transfected HEK293F cells were incubated with 100 µL lysis buffer (20 mmol/L Tris-buffered saline, 1% Triton X-100, 7.5 mL protease inhibitor cocktail (Sigma-Aldrich, Taufkirchen, Germany)) and 10 µL of 5% EDTA for 30 min at 4 °C and subsequently centrifuged at 10,000× *g* for 30 min. Protein concentration in cell lysates was determined and 30 µg of every lysate was electrophoresed under non-reducing conditions. Proteins were then transferred onto PVDF membrane (Millipore, Darmstadt, Germany) for immunoblotting and stained with moabs 7D8 (2 µg/mL) or anti-V5 tag (1 µg/mL; Clone TCM5; eBioscience^TM^ by Thermo Scientific, Waltham, MA, USA) in blocking buffer at 4 °C overnight. Bound antibodies were then visualised using HRP-labelled donkey anti-mouse IgG (Life Sciences, Waltham, MA, USA) and chemiluminescence substrate (Millipore, Burlington, MA, USA).

### 4.7. Immunoprecipitation of CD177

Cell lysates (350 µL, approximately 875 µg of total protein) or 10-fold concentrated cell culture supernatant (by use of Vivaspin20 columns (MWCO 3 kDa; Sartorius, Goettingen, Germany)) were incubated with 7D8 moab (3 µg) or mIgG as control (Ancell Corporation, Bayport, MN, USA) overnight at 4 °C. Next day, the antibody bound target proteins were incubated with protein G beads for one hour at room temperature. After incubation, the beads were washed, and bound proteins were eluted and separated by SDS-PAGE and subsequently detected with 7D8 and anti-V5 tag moabs.

### 4.8. Enzyme-Linked Immunosorbent Assay Using Recombinant CD177

Then, 96-well plates (Greiner Bio One, Kremsmuenster, Austria) were coated overnight with mab anti-V5 Tag (100 µL/well, Clone TCM5, 4 µg/mL; eBioscience^TM^ by Thermo Scientific, Waltham, MA, USA). Without washing, the plates were blocked for 1 h at room temperature with 100 µL/well in PBS/BSA 3%. After washing twice with 150 µL/well 0.2% PBS/BSA containing 0.05% Tween20, wells were incubated with the CD177 proteins (in duplicates). In each well, 100 µL of 10-fold concentrated cell culture supernatant containing recombinant human CD177 protein (wildtype and mutant form) was added. A culture supernatant of mock-transfected cells was used as a control. After incubation for 1.5 h at room temperature and washing (6×), 100 µL of 1:50 diluted human serum in washing buffer was added to each well and incubated for 1 h at room temperature. Next, plates were washed, and subsequently, 100 µL HRP labelled goat anti-human IgG (diluted 1:4000 in washing buffer; Jackson Immuno Research, West Grove, PA, USA) were added and incubated for 30 min at room temperature. In some experiments, to evaluate the binding of CD177 proteins to immobilised anti-V5, the bound proteins were then detected with HRP-labelled 7D8. After washing, 150 µL o-phenylenediamine in substrate buffer was added and incubated further for 10 min in the dark. The reaction was stopped with 50 µL 2.5 M H_2_SO_4_ and the plates measured at 492 nm in the ELISA plate reader (Tecan, Crailsheim, Germany). The reaction rate was calculated by dividing the optical density (OD) of the serum tested (against wildtype or *c.1291A*) against the control medium (without CD177 proteins). As a control, the reactivity of control sera (*n* = 20) with immobilised proteins was obtained. The cut-off was determined using the mean value of the reaction rates of control serums plus the threefold standard deviation. Serum sample reactivity against wildtype and *c.1291A* (*n* = 4) was compared by ANOVA analysis using GraphPad Prism 7.

### 4.9. Adsorption of Sera to CD177 Proteins

To evaluate the antibody combination contained in anti-HNA-2, 75 µL of serum was incubated with untransfected HEK293F (1 × 10^6^) at 37 °C for one hour with gentle mixing. Cells were centrifuged at 8000 rpm for one minute. For the second adsorption step, the supernatant was added to either wildtype or *c.1291A* mutant CD177 cells, or mock-transfected cells as control, and incubated for one hour at 37 °C. Cells were centrifuged at 8000 rpm for one minute, and supernatants were collected. Diluted (1:50) supernatant was used in ELISA as described above. In some experiments, sera were added to 100 µL 10-fold concentrated cell culture supernatant of CD177 wildtype or c.1291A or mock cells, mixed and incubated at 4 °C overnight. The next day, the samples were diluted 1:10 to reach a 1:50 dilution, which was directly added to immobilised CD177 (wild-type or mutant) in ELISA without any further purification. The adsorption efficiency of wildtype versus c.1291A protein was calculated based on average values of reactivity of each serum sample and adsorbate (*n* = 4). Statistical analysis was performed by ANOVA using GraphPad Prism 7.

### 4.10. Transient Co-Transfections of CD177 with PR3

Using the NEBuilder^®^ Hifi DNA Assembly Master Mix (New England Biolabs, Ipswich, MA, USA) cDNAs of CD177 full-length wild type or CD177 mutant form (c.1291G) were cloned into the vector pEGFP-c2 (Invitrogen, Waltham, MA, USA) and the cDNA sequence of proteinase 3 was cloned into pcDNA3.1/mCherry (Invitrogen, Waltham, MA, USA). COS-7 cells (ATCC, LGC Standards GmbH, Wesel, Germany) were cultured in DMEM medium (Capricorn Scientific, Ebsdorfergrund, Germany) supplemented with 10% foetal calf serum (FCS; PAN-Biotech, Germany) and Penicillin/Streptomycin (P/S; PAN-Biotech, Aidenbach, Germany). Using jetOPTIMUS (Polyplus-transfection, Illkirch, France), COS-7 cells were transfected with pEGFP-c2 (empty vector) or *pEGFP-CD177* c.1291G or *pEGFP-CD177* c.1291A together with pcDNA3.1/mCherry (empty vector) or pcDNA3.1/mCherry-PR3.

### 4.11. Confocal Laser Scanning Microscopy

The co-expression of CD177 (wildtype and mutant) with PR3 protein on transfected COS-7 cells was evaluated by confocal microscopy. Briefly, COS-7 cells were seeded on cover slips (High Precision Cover Glasses, 18 mm × 18 mm, 1.5H; Paul Marienfeld GmbH, Lauda-Koenigshofen, Germany) in 6 well plates (Greiner Bio One, Kremsmuenster, Austria) containing DMEM culture medium with 10% foetal calf serum (FCS; PAN-Biotech, Germany) and Penicillin/Streptomycin (P/S; PAN-Biotech, Aidenbach, Germany). Transfection was conducted and after at least 24 h, the medium was removed and the cells were washed three times with PBS+ (Capricorn Scientific, Ebsdorfergrund, Germany, with 1 mM CaCl_2_ and 0.5 mM MgCl_2_). Cells were fixed with a 4% formalin solution (Sigma-Aldrich, Taufkirchen, Germany; dilution of 10% stock solution with PBS, +Ca, and Mg) for 10 min at room temperature and washed. Cells were permeabilised using 0.2% Triton solution (0.2% Triton X-100 in PBS+, for 5 min at room temperature) and washed three times for 5 min and subsequently fixed. Cells were then carefully taken with a pair of tweezers and put on a slide with a drop of ProLong™ Diamond Antifade Mountant with DAPI (Invitrogen, Waltham, MA, USA) for about 24 h in the cold and the dark. The next day, cells were analysed with a Zeiss LSM 710 Confocal Laser Scanning Microscope (Carl Zeiss AG, Oberkochen, Germany). Data were obtained in ZEN 3.0 software (black edition, version 16.0.2.306 by Carl Zeiss AG, Oberkochen, Germany) and analyses were performed in GraphPad Prism 7 using ANOVA.

To investigate the interaction between CD177 and PR3 in living transfected cells, COS-7 cells were seeded on 60 mm cell culture dishes (Greiner Bio One, Kremsmuenster, Austria) containing a 30 mm cover glass, no. 1 (Paul Marienfeld GmbH, Lauda-Koenigshofen, Germany) and were transfected with the pEGFP-CD177 constructs (CD177 wildtype or c.1291A variant or empty vector) using 4 µg DNA and 4 µL jetOPTIMUS^®^ transfection reagent (Polyplus-transfection, Illkirch, France) as already described. For staining the nuclei, 24 h after transfection, the cells were incubated with a Hoechst 33,342 solution (dilution of stock solution 1:2000 in PBS; Thermo Scientific, Waltham, MA, USA) for 10 min in the dark. Cells were incubated at 37 °C and constantly syringed with BenchStable™ DMEM (1×) + GlutaMAX™-I (GIBCO BRL, Waltham, MA, USA) using a peristaltic pump system while imaging was conducted (Zeiss LSM 710 Confocal Laser Scanning Microscope, Carl Zeiss AG, Oberkochen, Germany).

The membrane stability of CD177 was investigated in live imaging of Hoechst 33342 stained EGFP-CD177 transfected COS-7 cells. Z-stacked images of living cells were cut into vertical slices and analysed using ZEN software. Co-localisation between CD177 (in both wildtype and mutant variants) and PR3 was calculated using correlation. Variance was calculated by R-Square (*R*^2^).

### 4.12. Statistical Analysis

Data were analysed using Prism 8 (GraphPad Software, Inc., San Diego, CA, USA). The co-localisation coefficient between mCherry and EGFP in transfected cells expressing CD177 wildtype or mutant and PR3 was analyzed by ANOVA.

## 5. Conclusions

In conclusion, the results of this study change and further add to the mechanism regulating CD177 absence from the surface of neutrophils. The presence of *CD177* c.1291A leads to the production of an unstable CD177 protein which is removed from the neutrophil surface and therefore leads to an apparent “CD177-null” phenotype. Similar to the wildtype form, soluble *CD177* c.1291A appears to carry all relevant anti-HNA-2 epitopes and binds to PR3.

## Figures and Tables

**Table 1:** Calculation of PR3 co-localisation with *CD177* c. 1291G (wildtype) or *CD177* c.1291A (mutant) in transfected cells.

	EGFP-*CD177* c.1291G/ mCherry-PR3	EGFP-*CD177* c.1291A/ mCherry-PR3	EGFP/ mCherry	EGFP/ mCherry-PR3
(Weighted) Co-localisation Coefficient EGFP	0.619 (0.713)	0.682 (0.754)	0.484 (0.518)	0.059 (0.086)
(Weighted) Co-localisation Coefficient mCherry	0.789 (0.840)	0.791 (0.998)	0.618 (0.673)	0.873 (0.885)
Overlap Coefficient	0.95	0.95	0.83	0.91
Correlation R	0.69	0.66	0.10	0.32
Correlation RxR	0.52	0.50	0.11	0.16

The correlation R as a mathematical description of the dependence (without conclusive need of causality; −1–1) as well as the correlation R2 (explanation of causality; values until +1) were generated. Representative results of three independent experiments. The measured fluorescence signals of 20 cells from each transfection were analysed in GraphPad Prism 7 using ANOVA.

## Data Availability

Data is contained within the article and Supplementary Materials.

## Supplementary Materials

The following supporting information can be downloaded at: https://www.mdpi.com/article/10.3390/ijms25052877/s1.
