# Supplementary material for: The CD177 c.1291A Allele Leads to a Loss of Membrane Expression and Mimics a CD177-Null Phenotype
Source: Int J Mol Sci. 2024 Mar 1;25(5):2877. doi: 10.3390/ijms25052877 (PMC10931634; doi:10.3390/ijms25052877)
Supplement: Supplementary file 1 [file ijms-25-02877-s001.zip › ijms-2532489-supplementary.pdf]

Supplementary materials:

Prediction of GPI-modification

To predict the effect of CD177 c.1291G>A polymorphism, the protein sequence of both CD177 variant (wildtype and mutant) were analysed in big-PI predictor GPI modification site and GPI-anchor possiblities for both proteins were scored.

MSAVLLLALL GFILPLPGVQ ALLCQFGTVQ HVWKVSDLPR
QWTPKNTSCD SGLGCQDTLM LIESGPQVSL VLSKGCTEAK
DQEPRVTEHR MGPGLSLISY TFVCRQEDFC NNLVNSLPLW
APQPPADPGS LRCPVCLSME GCLEGTTEEI CPKGTTHCYD
GLLRLRGGGI FSNLRVQGCM PQPVCNLLNG TQEIGPVGMT
ENCDMKDFLT CHRGTTIMTH GNLAQEPTDW TTSNTEMCEV
GQVCQETLLL LDVGLTSTLV GTKGCSTVGA QNSQKTTIHS
APPGVLVASY THFCSSDLCN SASSSSVLLN SLPPQAAPVP
GDRQCPTCVQ PLGTCSSGSP RMTCPRGATH CYDGYIHLSG
GGLSTKMSIQ GCVAQPSSFL LNHTRQIGIF SAREKRDVQP
PASQHEGGGA EGLESLTWGV GLALAPALWW **G**VVCPSC

Total Score for Potential GPI.....................: **5.50**

MSAVLLLALL GFILPLPGVQ ALLCQFGTVQ HVWKVSDLPR
QWTPKNTSCD SGLGCQDTLMLIESGPQVSL VLSKGCTEAK
DQEPRVTEHRMGPGLSLISYTFVCRQEDFCNNLVNSLPLWAPQ
PPADPGSLRCPVCLSMEGCLEGTTEEICPKGTTHCYDGLLRLRG
GGI FSNLRVQGCMPQPVCNLLNGTQEIGPVGMTENCDMKDFLT
CHRGTTIMTH GNLAQEPTDW TTSNTEMCEVGQVCQETLLL
LDVGLTSTLV GTKGCSTVGA QNSQKTTIHS APPGVLVASY
THFCSSDLCNSASSSSVLLN SLPPQAAPVP GDRQCPTCVQ
PLGTCSSGSP RMTCPRGATH CYDGYIHLSGGGLSTKMSIQ
GCVAQPSSFL LNHTRQIGIF SAREKRDVQP PASQHEGGGA
EGLESLTWGVGLALAPALWW **R**VVCPSC

Total Score for Potential GPI.....................: **-6.98**

Supplementary Figure S1: Prediction of GPI modification induced by the CD177 c.1291G>A polymorphism (bold letter) by big-PI Predictor GPI Modification Site. The total score for GPI in the wild type CD177 (left) and the mutant variant (right) is calculated by the big-PI Predictor Modification Software.
